# Irisin Association with Ki-67, MCM3 and MT-I/II in Squamous Cell Carcinomas of the Larynx

**DOI:** 10.3390/biom12010052

**Published:** 2021-12-30

**Authors:** Agnieszka Pinkowska, Katarzyna Nowinska, Urszula Ciesielska, Marzenna Podhorska-Okolow

**Affiliations:** 1 Division of Anatomy, Department of Human Morphology and Embryology, Wroclaw Medical University, 50-368 Wroclaw, Poland; agnieszka.pinkowska@umw.edu.pl; 2 Division of Histology and Embryology, Department of Human Morphology and Embryology, Wroclaw Medical University, 50-368 Wroclaw, Poland; urszula.ciesielska@umw.edu.pl; 3 Division of Ultrastructural Research, Wroclaw Medical University, 50-368 Wroclaw, Poland; marzenna.podhorska-okolow@umw.edu.pl

**Keywords:** irisin, Ki-67, MCM3, MCM5, MCM7, MT-I/II, LSCC

## Abstract

Background: Current studies indicate irisin role in carcinogenesis. The aim of the study was to investigate the expression of irisin in LSCCs and to determine its association with clinicopathological factors, as well as recognized markers of proliferation, i.e., Ki-67 and MCM3,5,7 and MT-I/II proteins. Material and methods: The research material consisted of 140 cases of LSCCs, 57 cases of laryngeal papillomas (BLs) and 14 controls (benign hypertrophic changes). Tissue microarrays were used to perform IHC. Western blot and immunofluorescence were performed in laryngeal cancer cell lines and normal keratinocytes. Results: Irisin expression levels were significantly increased in LSCC compared to BLs (*p* < 0.0001) and controls (*p* = 0.001). We noted a positive moderate and weak correlation between irisin and Ki-67, MCM3 and MT-I/II. We observed an elevated level of irisin expression with increasing tumor size (T1–2 vs. T3–4; *p* = 0.0348). The levels of irisin were higher in N0 than in N1 and N2–3 (*p* = 0.0031 and *p* = 0.0457, respectively). Our in vitro study revealed a higher level of irisin in Larynx Epidermoid Carcinoma 2 (HEp-2) cells compared to the control Normal Human Keratinocyte (HaCat) cell line. Conclusions: Increased irisin expression levels in LSCC and its correlation with clinicopathological and proliferation factors may indicate the potential role of irisin as a biomarker in the diagnostic process of LSCC.

## 1. Introduction

The larynx is the most common localization of head and neck cancers, and the most prevalent type is squamous cell carcinoma (SCC), which is diagnosed in 95% of cases [1]. The risk of laryngeal cancer is eight times more frequent in men. In the male population, laryngeal cancer is the seventh most common cancer [2]. Total laryngectomy still remains the gold standard of therapeutic management due to the lack of specific methods for the diagnosis of laryngeal cancer [3,4]. Radical surgery is associated with the loss of function of the larynx, depriving patients of the possibility of communication, which has a very negative impact on their functioning and recovery [5,6]. Due to the difficulties associated with the diagnosis of laryngeal cancer, there is a necessity to search for specific markers related to carcinogenesis in this area. Irisin could be one such marker. The increased level of irisin expression has been reported in many cancers. Its elevated level of expression has been observed, for example, in thyroid and lung cancer [7,8,9]. The anatomic proximity of the thyroid and larynx, as well as the location of the larynx on the border between the upper and lower respiratory tract raise the question about the potential role of irisin in the carcinogenesis of laryngeal cancer. The expression of irisin in laryngeal carcinomas has not been studied yet.

Irisin was described as a myokine released by skeletal muscle in response to physical exercise in 2012. Bostrom et al. [10] observed that irisin was released as a result of physical exercise and affected white adipose tissue (WAT) cells, which turn into beige. Phenotypically and functionally, they resemble brown adipose tissue (BAT). Irisin is released as a result of post-translational modifications of the FNDC5 prohormone, encoded by the FNDC5 gene. The peptide composed of 112 amino acids is the result of cleavage and glycosylation of the extracellular domain of the prohormone [11,12]. FNDC5 is an N-glycan with two potential glycosylation sites (Asn36 and Asn81) [13]. Glycosylation is one of the most important post-translational modifications affecting the physicochemical properties of protein [14]. Inhibition of glycosylation decreases irisin secretion [13] and also increases its molecular mass from 13 kD to 20 kD [11]. The increase in FNDC5 expression under the influence of the peroxisome proliferator-activated receptor gamma coactivator 1-alpha (PGC1-α) elevates the expression of the BAT uncoupling protein 1 (UCP1) marker. The effect of thermogenin activity is energy dissipation, which has a positive effect on metabolism, and may prevent obesity and related diseases, including type II diabetes [15,16]. In addition to muscle and adipose tissue, many studies have shown the presence of irisin in other tissues and organs, such as the brain, testes, epididymis, heart muscle cells, skin, liver, pancreas, and stomach [17]. Moreover, the study showed the impact of irisin on tissues as a ligand of integrin receptor [18,19,20].

Irisin expression was also observed in many types of cancer, including breast, prostate, gastrointestinal tract, bone, and lung cancer as well as gynecological carcinomas [21,22,23,24]. In the case of non-small-cell lung carcinoma (NSCLC), the presence of irisin has been noted not only in cancer cells but also in cancer-associated fibroblasts (CAFs). The elevated level of irisin expression in CAFs was associated with a worse prognosis in patients and the proliferation of NSCLC cells [8]. Moreover, CAFs participate in carcinogenesis by stimulating neoplastic angiogenesis and the epithelial–mesenchymal transition (EMT) [25]. Other studies have revealed that irisin may be involved in limiting neoplastic expansion. Irisin was shown to inhibit EMT by blocking PI3K/Akt/Snail signaling, affecting the inhibition of migration and invasion of lung cancer cells in an in vitro model [9]. In addition, many studies on the in vitro model revealed that irisin also inhibited cell proliferation, for example, in breast, lung, bone, prostate, and pancreatic gland cancer [9,22,24,26,27]. Their findings are contrary to the studies that showed that irisin did not affect the proliferation, adhesion, or colony formation of malignant cells of the colon, endometrium, thyroid, or esophagus [28]. 

The influence of irisin on the proliferative activity of cancer cells seems to be of significant importance for its potential role in the diagnostic process. Therefore, in our study, we estimated the correlation with recognized markers of proliferation, such as Ki-67 antigen and minichromosome maintenance proteins (MCM3, MCM5, and MCM7) [29]. Ki-67 is a non-histone protein in the cell nucleus that is responsible for cell proliferation. In LSCC cancers, a significant correlation was demonstrated between the tumor grade (G) and the Ki-67 proliferation index value [30,31]. However, the MCM family includes proteins from MCM1 to MCM10. The basic function of MCM proteins is participation in the mechanism of initiation of replication and regulation of DNA synthesis. MCM proteins play an important role in maintaining genome integrity by preventing DNA from another replication in the same cell cycle. MCM proteins are not expressed in the resting phase (G0). However, they are detected during all other phases (G1, S, G2 and M) and can, therefore, serve as potential markers of cell proliferation and can also be useful to assess the degree of tumor cell proliferation [32]. Studies showed an increased expression of MCM5 and a positive correlation with Ki-67 [33], as well as an increased expression of MCM2, MCM3, and MCM7 in Larynx Epidermoid Carcinoma 2 (HEp-2) cell lines and positive correlations with Ki-67 in LSCC [34]. In addition, we decided to investigate the correlation of irisin with metallothioneins I and II (MT-I/II). MT-I/II are low-molecular-weight proteins that are characteristic of most tissues. They participate in the neutralization of free oxygen radicals and the homeostasis of many chemical elements, and play an important role in the detoxification of heavy metals [35]. They are also involved in carcinogenesis and stimulate cell proliferation and angiogenesis. The increase in MT concentration in the cell correlates with the tumor grade. MTs are involved in the immune response, wound healing, and the development of multidrug resistance (MDR) [36]. The importance of MT expression as a tumor biomarker is mainly observed in squamous neoplasms of the larynx [34], lung [37], stomach, intestine, pancreas, uterus, and breast [38].

Studies on irisin expression in neoplastic tissues have demonstrated its potential as an independent diagnostic and prognostic factor. The expression of irisin in laryngeal cancer has not been studied yet. Therefore, the aim of the study was to determine the localization and the level of irisin expression in LSCC, as well as to investigate its relationship with such markers as Ki-67, MCM3,5,7, or MT-I/II and with clinicopathological factors in LSCC.

## 2. Materials and Methods

### 2.1. Patients Cohort

The study was conducted on material consisting of 140 cases of laryngeal squamous cell carcinomas (LSCCs) and 57 cases of laryngeal papillomas (BLs), which were collected between 1997 and 2003. All patients were diagnosed and treated at the Department of Pathomorphology of the J. Babinski Regional Hospital of Wroclaw and the Department and Clinic of Otolaryngology, Head and Neck Surgery, Wroclaw Medical University. Fourteen blocks with the sections of vocal cord nodules and Reinke’s edema were used as the control. Patients gave their written informed consent, and the study was approved by the Bioethical Committee of Wroclaw Medical University (ID No. KB-355/2019). The mean age of the patients during the treatment period was 59 years (41–79 years). The treatment and follow-up included 20 women and 120 men based on the TNM classification of the International Union Against Cancer (UICC) [39], the grade of malignancy (G), and the clinical stage of LSCC were determined.

### 2.2. Cell Line Culture

Protein expression studies (Western blot and immunofluorescence [IF]) were conducted using the reference Larynx Epidermoid Carcinoma 2 (HEp-2) (collection of cell lines of the Ludwik Hirszfeld Institute of Immunology and Experimental Therapy, Polish Academy of Sciences, Wroclaw, Poland) adherent laryngeal cancer cell line, and the Normal Human Keratinocyte cell line (HaCaT) (The American Type Culture Collection, Manassas, VA, USA), an adult normal immortalized human keratinocyte cell line, was used as the control. HEp-2 cells were cultured in EMEM medium (Lonza, Basel, Switzerland). HaCaT cells were cultured in DMEM medium (Lonza). Both media were supplemented with 10% fetal bovine serum (FBS) (Merck, Darmstadt, Germany), 1% penicillin/streptomycin (Merck), and L-glutamine (Merck). The HERA cell incubator (Heraeus, Hanau, Germany) was used to maintain constant conditions of cell cultures, i.e., a temperature of 37 °C, 5% CO_2_ concentration, and a 95% humidity level.

### 2.3. Tissues Microarray (TMA) Preparation

Tissue sections were fixed in 10% buffered formalin, dehydrated, and embedded in paraffin. Tissue microarrays (TMA) were performed from paraffin blocks—1 TMA containing the material with vocal cord nodules, 5 TMAs containing LSCC tumors, and 2 TMAs with BLs. Routinely, paraffin blocks were cut and hematoxylin and eosin (HE) staining was performed. The HE stained slides were examined under light microscopy (BX-42; Olympus, Tokyo, Japan) by two independent pathologists. Subsequently, the HE slides were scanned using the Pannoramic Midi II histological scanner (3DHistech, Budapest, Hungary). Using the Pannoramic Viewer Program (3DHistech), representative cancers sites with a core size of 1.5 mm were selected, followed by their transfer to the tissue recipient arrays using the TMA Grand Master (3DHistech Ltd., Budapest, Hungary).

### 2.4. Immunohistochemistry (IHC)

TMAs were cut into 4-µm-thick paraffin sections and mounted on Superfrost Plus slides (Menzel Gläser, Braunschweig, Germany). Deparaffinization, hydration, and thermal demasking of epitopes were performed using the Pre Treatment Link Station (Dako, Glostrup, Denmark). The slides were incubated at 97 °C for 20 min with the Target Retrieval Solution in high-pH buffer (Agilent Technologies, Santa Clara, CA, USA). IHC reactions were performed using specific polyclonal anti-irisin rabbit antibodies (dilution 1:50; code no. NBP2-14024; Novus Biologicals, Littleton, CO, USA), mouse monoclonal antibodies: anti-MCM5 (clone E-10, dilution 1:100, Santa Cruz Biotechnology, Dallas, TX, USA), anti-Ki-67 (clone MIB1, ready to use; Agilent Technologies), anti-MT-I/II (clone E9, dilution 1:100; Agilent Technologies), anti-MCM2 (clone CRCT2.1, dilution 1:15; Leica Biosystems, Nussloch, Germany), anti-MCM3 (clone 101, dilution 1:50; Agilent Technologies), and anti-MCM7 (clone DCS-141.1, dilution 1:50; Leica Biosystems). The antibody diluent (Agilent Technologies), with a background reducing component, was used to dilute the primary antibodies. IHC reactions were performed using the Autostainer Link (Agilent Technologies) and the EnVision™ FLEX high-pH Link visualization system (Agilent Technologies). Control of the immunohistochemical reaction was performed without the addition of the primary antibody.

### 2.5. Evaluation of Immunohistochemistry (IHC)

A positive IHC reaction of irisin, Ki-67, MCM3,5,7, and MT-I/II in tissue specimens in LSCCs, BLs, and control was observed using the BX41 light microscope (Olympus, Tokyo, Japan) coupled with the Cell D program (Olympus). The analysis of the expression of the proteins was performed using ×200 magnification. The intensity of the cytoplasmic reaction detecting the presence of irisin and of the MT-I/II semiquantitative Immunoreactive Score (IRS) method, according to Remmele and Stegner [40], were used. 

The evaluation of the IHC reaction was conducted by two independent pathologists. The intensity of the color reaction in the cytoplasm of cells (from 0 to 3 points) and the percentage of neoplastic cells with a positive reaction (from 0 to 4 points) were estimated. The score is the result of the multiplication of color intensity and percentage of cells with positive reactions. The result can take values from 0 to 12 points. Details of the IRS scale are presented in Table 1.

The percentage of cells with a positive reaction was calculated compared to the total number of tumor cells. The level of Ki-67, MCM3, MCM5, and MCM7 expression was assessed with the use of a five-point scale (0 point—>0%; 1 point—>0–10%; 2 points—>10–25%; 3 points—>25–50%; 4 points—>50–100% of expression) [41,42].

### 2.6. Immunofluorescence (IF)

To perform IF, 24-h microculture cells were placed on slides (Millicell EZ 8-well glass slides; Merck). For microculture, 600 μL of 20 × 10^4^ cells/mL suspension was instilled into each well on the slides. Microcultures with cells were placed in an incubator at 37 °C for 24 h. After the incubation, the cells were fixed with the use of 4% formaldehyde and further incubation was conducted with the specific polyclonal rabbit anti-irisin/FNDC5 antibody (dilution 1:50; code no. NBP2-14024; Novus Biologicals) at 4 °C overnight. After rinsing, the slides were incubated for 1 h with polyclonal donkey anti-rabbit secondary AlexaFluor 568 conjugated antibody (dilution 1:2000; code no. A10042; Invitrogen, Carlsbad, CA, USA) in the reagent with a background-reducing component (Agilent Technologies). The slides were mounted using the Prolong DAPI Mounting Medium (Invitrogen). The observations were made at x600 magnification with the use of Fluoview FV3000 confocal microscopy (Olympus) coupled with CellSense software (Olympus, RRID:SCR_016238).

### 2.7. Western Blot Analysis

For each analysis, 3 × 10^6^ HEp-2 and HaCat cells were taken. The extraction of whole cell proteins was made using the RIPA buffer (50 mM Tris HCl; 150 mM NaCl; 0.1% SDS; 1% Igepal (CA-630, Merck, Darmstadt, Germany); 0.5% sodium deoxycholate; protease inhibitor cocktail (Merck); 0.5 mM PMSF). The concentration of proteins was determined using the Pierce BCA Protein Assay Kit (Thermo Fisher Scientific, Waltham, MA, USA). The proteins were denatured in sample loading buffer (250 mM Tris-HCl, 40% glycerol, 20% β-mercaptoethanol, 8% SDS and bromophenol blue), transferred to a PSQ membrane (Millipore, Burlington, MA, USA), and blocked with 2% non-fat milk (Bio-Rad, Marnes-la-Coquette, France) in 0.1% TBST for 1 h at room temperature. Next, the membrane was incubated with rabbit polyclonal anti-irisin/FNDC5 antibody diluted in 0.5% milk in 0.1% TBST (1:200, code no. NBP2-14024, Novus Biologicals) overnight at 4 °C with gentle shaking. Incubation with the secondary horseradish peroxidase, conjugated with donkey anti-rabbit antibody and diluted in 0.5% milk in 0.1% TBST (1:3000, code no. 711-035-052; Jackson ImmunoResearch, Cambridgeshire, UK), was performed for 1 h at room temperature. The proteins were visualized using the Luminata Classico Western HRP Substrate (Millipore). The membrane was stripped and incubated again with monoclonal mouse anti-βactin antibody diluted in 1% milk in 0.1% TBST (dilution 1:5000, clone 2D4H5, code no. 66009-1-Ig, Proteintech, Rosemont, IL, USA), and this was used as the control for the amount of protein loading. The data were collected in the Chemi-Doc XRS Molecular Imager apparatus (Bio-Rad). The optical density of the protein band was measured with the use of the Image Lab (Bio-Rad) software. The experiment was repeated three times.

### 2.8. Statistical Analysis

The Kolmogorov–Smirnov test was used to check the normality of the distribution. The differences in the expression of irisin in BLs and LSCCs, as well as their relationship with clinicopathological factors, were examined using the Kruskal–Wallis test or the Mann–Whitney U test. Associations between irisin and the MCM2, MCM3, and MCM7 proteins, Ki-67 antigen, and MT-I/II were assessed by the Spearman rank correlation test. The Kaplan–Meier analysis and the Cox regression method were used to check the relationship between the intensity of irisin expression and overall survival. The unpaired t-test was used to assess the differences between the level of irisin in cell lines. The statistical analysis was performed using Prism 5.0 (GraphPad, La Jolla, CA, USA) software and *p* values < 0.05 were considered statistically significant. 

## 3. Results

### 3.1. Immunohistochemical (IHC) Detection of Irisin Expression in Tissue Microarrays (TMAs)

**Figure 1:**
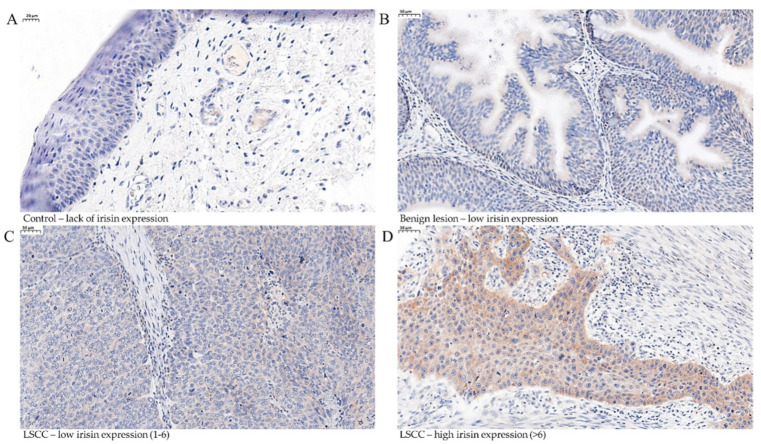
Immunohistochemical (IHC) reactions (brown) indicating irisin expression in the cytoplasm performed on control tissue (**A**) (magnification ×400, scale bar 20 μm, benign lesions (BLs)) and (**B**) in laryngeal squamous cell cancer (LSCC) (low (1–6) (**C**) and high (>6) (**D**)) (magnification ×200).

**Figure 2:**
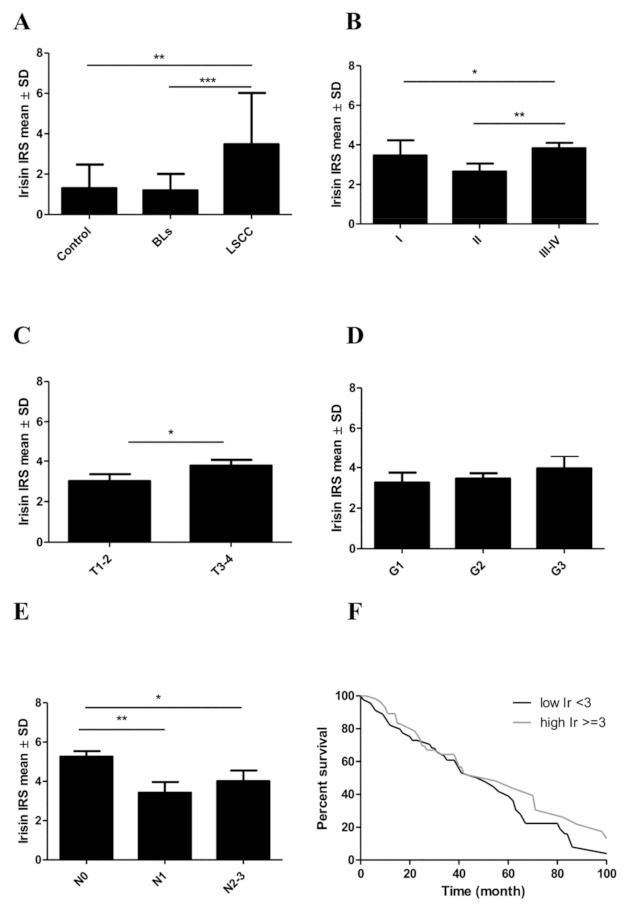
Comparison of irisin expression levels detected by immunohistochemistry (IHC) in control tissues, benign lesions (BLs), and laryngeal squamous cell cancer (LSCC) (**A**). Comparison of irisin expression levels in LSCC cells according to the tumor stage (**B**), tumor size (**C**), the grade of malignancy (**D**), and the lymph node status (**E**). Kaplan–Meier survival curves show the prognostic impact of irisin expression levels on overall survival (OS) of patients with LSCC. Patients were grouped according to the median value of expression levels (**F**), * *p* ≤ 0.05, ** *p* ≤ 0.005, *** *p* ≤ 0.001.

Our study showed the expression of irisin in controls, BLs, and LSCCs (Figure 1). In LSCC, irisin expression was found in the cytoplasm of cancer cells. No significant difference of irisin expression was found between the control group and BLs. However, irisin expression levels were significantly increased in LSCCs compared to BLs (Mann–Whitney U test, *p* < 0.0001), and the control group (Mann–Whitney U test, *p* = 0.001) (Figure 2A). The associations between low and high expression and clinicopathological characteristics of patients are shown in Table 2.

### 3.2. Associations between Irisin Expression in Cancer Cells and Clinicopathological Parameters

The relationships between the level of irisin expression in cancer cells and the clinical and pathological parameters in LSCC are given in Table 3. The mean level of irisin expression for stage I was 3.46 ± SD 1.86, then it decreased to 2.65 (±SD 2.52) in stage II and increased again in stages III and IV (mean 3.86 ± SD 2.55). We observed a statistically significant difference between stage II and stages III–IV (Mann–Whitney U test, *p* = 0.0083) (Figure 2B).

We also observed an elevated level of irisin expression with increasing tumor size (T). The differences between T1–2 and T3–4 were statistically significant (Mann–Whitney U test, *p* = 0.0348). The highest mean level of irisin was observed in T3–4 (mean 3.78 ± SD 2.58) compared to T1–2 (mean 3.00 ± SD 2.45) (Figure 2C). The level of irisin expression in LSCC increased slightly with the increase in tumor malignancy (G). The differences between the individual groups were not statistically significant (Figure 2D).

We also noticed an association between irisin expression levels and lymph node metastasis. The highest mean value was observed for N0 (5.25 ± SD 2.02) and the lowest for N1 (mean 3.41 ± SD 2.10). Finally, the mean value (4.00 ± SD 2.68) increased again with lymph node metastasis (N2–3). The differences between the levels of irisin in N0 and N1 and in N0 and N2–3 were statistically significant (Mann–Whitney U test, *p* = 0.0031 and *p* = 0.0457, respectively) (Figure 2E). We did not observe any association between overall survival (OS) and irisin, MCM3,5,7, and Ki-67 in patients with LSCC (Table 4, Figure 2F). 

### 3.3. Associations between Irisin and Cancer Cell Proliferation

**Figure 3:**
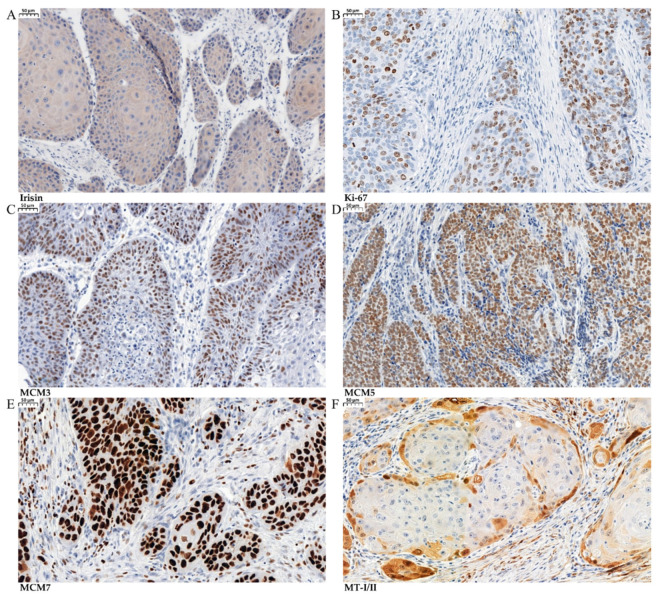
Immunohistochemical (IHC) reactions (brown) indicating irisin expression in the cytoplasm in laryngeal squamous cell cancer (LSCC) (**A**) with antigen Ki-67 (**B**), MCM3 (**C**), MCM5 (**D**), MCM7 (**E**), and MT-I/II (**F**) in LSCC (magnification ×200).

The expression of other proteins (Ki-67, MCM3,5,7) that are associated with proliferation was observed in nuclei, and the expression of MT-I/II was found in the cytoplasm (Figure 3A–F). 

**Figure 4:**
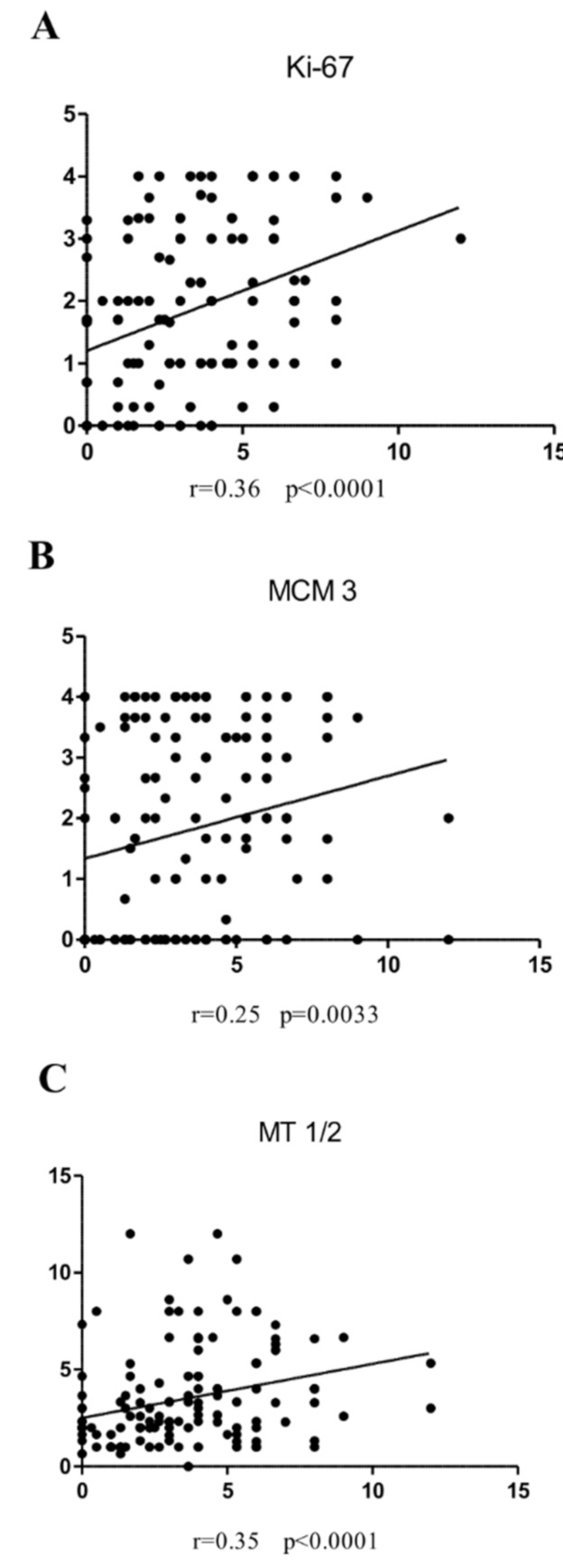
Correlations of irisin expression levels with diagnostic markers are moderate positive Ki-67 (**A**), weak positive MCM3 (**B**), and moderate positive MT-I/II (**C**) in LSCC cells.

We found a positive moderate correlation between irisin and the Ki-67 antigen expression level in the nuclei of LSCC cells (r = 0.36; *p* < 0.0001) (Figure 4A). In addition, we identified a weak positive relationship between irisin and MCM3 expression levels (r = 0.25; *p* = 0.0033) (Figure 4B). However, we did not observe any significant correlation between irisin and MCM5 expression (r = 0.12, *p* = 0.1512), or between irisin and MCM7 (r = −0.17, *p* = 0.790). A moderate positive correlation was also demonstrated by comparing the level of irisin expression in LSCC and MT-I/II (r = 0.35, *p* < 0.0001) (Figure 4C).

### 3.4. Irisin Expression Levels in Cancer Cell Lines

**Figure 5:**
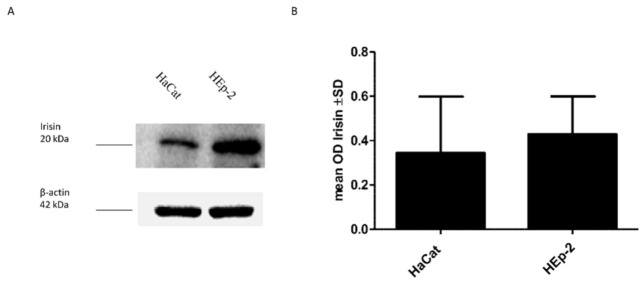
Comparison of irisin expression levels detected by Western blot in Normal Human Keratinocytes (HaCat) and Larynx Epidermoid Carcinoma 2 (HEp-2) (**A**). The mean value of the densitometric optical analysis of the 3 repeats of Western blot detecting irisin expression levels (**B**).

**Figure 6:**
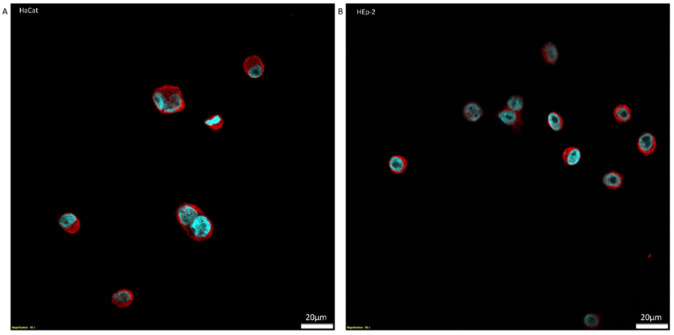
Localization of irisin (red color) around the nucleus (blue color) expression in cells’ cytoplasm detected by immunofluorescence reaction (IF) in Normal Human Keratinocytes (HaCat) (**A**) and Larynx Epidermoid Carcinoma 2 (HEp-2) (**B**) (magnifications ×600, scale bar-20 μm (lower right corner)).

Our in vitro study indicated the presence of irisin expression in both cell lines. However, a higher level of irisin was observed in HEp-2 laryngeal cancer cells compared to its level in control HaCat cells. The difference was not statistically significant. The results obtained using Western blot (Figure 5) were confirmed by confocal microscopy (Figure 6).

## 4. Discussion

Our study is the first to assess irisin expression in LSCC tissues. The investigation showed the expression of irisin in both non-cancerous and LSCC cells. A significantly higher concentration of irisin was observed in LSCC with regard to BLs and controls. Irisin was expressed primarily in the cytoplasm of laryngeal cancer cells. Previous studies were focused on the distal part of the respiratory tract. It was also the first study that used the tissue obtained from NSCLC tumors and the results were correlated with the clinicopathological data of patients [8]. Former studies confirming the expression of irisin in cancer cells of the respiratory system were carried out in an in vitro model using lung cancer cell lines [9]. An increased irisin expression in cancer cells of the head and neck area was observed in the tissues of thyroid tumors [7]. Ugur et al. [7] also showed that the increased concentration of irisin could be used in the differential diagnosis between malignant tumors and benign hyperplastic changes in the thyroid gland. Among other tumor tissues, increased irisin expressions were observed in cancers of the breast, ovary, cervix [21], and gastrointestinal tract [23], with the exception of hepatocellular carcinoma. In contrast, decreased irisin levels were observed in renal cancer tissues [43].

Most studies on irisin levels in cancers were based on its detection in the serum of patients. It was not possible to determine whether local or systemic production was responsible for the increase in irisin concentration in tumor cells. The results of the tests using ELISA determining the serum concentration of irisin were inconclusive compared to the control group. Irisin levels were higher in patients with renal cancer [44], lower in patients with breast cancer [45], and unchanged in subjects with hepatocellular carcinoma [46]. A high expression of irisin was observed in skeletal muscles, whereas a moderate expression was found in adipose tissue [47]. However, the probability of irisin production by skeletal muscles and adipose tissue in cancer patients was reduced by their physical condition, and often by progressive cachexia. A decreased level of irisin in circulating blood could be the result of increased local production and a signal for the systemic regulation [45].

Moreover, in our studies, irisin expression levels were compared with clinicopathological parameters. We observed a higher irisin expression in larger tumors (T3–4) and tumors with a high grade of malignancy (G3). However, in the case of malignancy grade, we did not observe statistical significance. However, Panagiotou et al. [48] observed an increase in serum irisin levels in higher grades (Elston-Ellis) and the differences were significant, which confirmed our observation. Tumor growth requires a lot of energy and glucose, which are crucial for tumor proliferation. The consumption of glucose by cancer cells through aerobic respiration increases [8] and the overexpression of membrane glucose transporters (GLUTs) occurs. Overexpression and translocation to the membrane of GLUTs have been observed in many cancers, including breast, large intestine, salivary gland, and stomach cancer [49]. There is evidence that irisin affects GLUT4 translocation in the skeletal muscle of diabetic mice. Studies showed that irisin increased glucose tolerance and uptake. Irisin also improved glucose metabolism by increasing the phosphorylation of 5′AMP-activated protein kinase (AMPK) in myocytes and hepatocytes in in vitro and in vivo studies [50]. Irisin may change the metabolism of cancer cells, which could explain its relationship with the increase in its proliferation. However, further studies are warranted to explain this mechanism. 

Most studies performed on the in vitro model revealed the inhibitory effect of irisin on the proliferation of cancer cells [9,27,28]. However, there are no studies that determined the existence of such a relationship in neoplastic tissues. Considering the results of these studies and the lack of knowledge in this area, we decided to investigate the correlation of irisin expression in LSCC with the standard markers of cancer cell proliferation. We obtained a positive moderate and weak correlation with Ki-67, MCM3, and MT-I/II antigens, which could indicate the potential role of irisin in the proliferation of LSCC cells. Positive correlations of irisin expression with cellular proliferation markers were also reflected and confirmed by association with clinicopathological factors (G, pT, pN, and Stage) in LSCC. Panagiotou et al. [48] also observed a positive correlation of serum irisin levels with the Ki-67 antigen in patients with benign and malignant breast cancer. Moreover, Shi et al. [46] reported that in human hepatocellular carcinoma (HHC), irisin could enhance the proliferation of neoplastic cells via the PI3K/AKT pathway. Contrary to our results, Shao et al. [9] indicated that a higher level of expression could lead to the inhibition of proliferation, migration, and the epithelial–mesenchymal transition (EMT). However, these studies used an in vitro model. In our previous study, we observed associations between irisin and cancer cell proliferation in NSCLC cells. Irisin expression in cancer cells correlated negatively with the expression of the Ki-67 antigen in NSCLC cells [8]. The differences may be due to tissue specificity and, hence, further research is warranted. 

The phenomenon of EMT is the basis of metastasis [51]. Previous studies showed that irisin could be involved in cancer metastasis by inhibiting the EMT [24,27]. In our study, we observed the highest expression of irisin in the group of patients without lymph node metastasis (N0). This could demonstrate the protective effect of irisin in the pre-metastatic tumor. A similar protective effect of high levels of irisin (serum) was observed in breast cancer. The study on patients with breast cancer revealed that serum irisin level was higher in the group without spinal metastasis. The patients with higher serum levels of irisin had a 20% reduction in the possibility of metastasis [47]. In our opinion, the increased level of irisin in the N0 group of patients is associated with the inhibition of EMT and the prevention of metastasis in LSCC. So far, only a few studies have analyzed the possible relationship between irisin expression and EMT. The results of these studies suggest that irisin may reverse the EMT process of cells. Kong et al. [24] observed that irisin reverses EMT depending on the IL-6 pathway in osteosarcoma. They suggest that irisin regulates Snail expression via STAT3. In our study, the level of irisin decreased in patients who had metastasis to a single lymph node on the same side of the neck as the tumor (N1). The level of irisin increased again if cancer cells spread to more lymph nodes (N2–3). Despite the re-increase in irisin levels in N2–3, it did not reach a level as high as that of N0. The explanation of the re-increase in irisin expression in the N2–3 group requires further research in order to enable full understanding of this phenomenon.

Our IHC study revealed a higher irisin expression in LSCC cells compared to control tissues. The levels of irisin expression determined by Western blot and IF were the same as expected. In the cell lines, irisin expression was significantly higher in the laryngeal cancer cell line (HEp-2) compared to the control cell line (HaCat). IHC confirmed the results obtained in the in vitro model. Our previous study on lung cancer cells also indicated the higher expression of irisin in cancer cells compared to control cells [8]. Shi et al. [46] showed increased proliferation, migration, and invasiveness of hepatocellular carcinoma cells caused by activation of the PI3K/Akt pathway after irisin treatment. On the other hand, Moon et al. [28] showed the inhibiting effect of irisin treatment on the proliferation of endometrial (KLE and RL95-2), colon (HT29 and MCA38), thyroid (SW579 and BHP7), and esophageal (OE13 and OE33) cancer cell lines. The ambiguous results of the studies may arise from the tissue and cell specificity in irisin expression [46] or may be associated with the form of irisin used (glycosylated or non-glycosylated) [9,24,26,27,28,46]. The studies in the in vitro model investigating the effects of irisin on cancer cells and the expression levels in cancer cells showed unclear results. More research is warranted to determine the effects of irisin on cancer cells.

## 5. Conclusions

In conclusion, our study was the first to show irisin expression in LSCC. We observed a higher irisin expression for LSCC compared to control tissues and its association with tumor growth and lymph node metastasis. Moreover, we analyzed irisin expression with clinicopathological factors and established cancer proliferation factors, indicating the potential role of irisin as a biomarker in the diagnostic process of LSCC.

## Figures and Tables

**Table 1:** The semiquantitative scale assessing the irisin and MT-I/II expression in control tissues of the larynx, BLs, and LSCC according to Remmele and Stegner (IRS) [40]. Modified table according to Nowinska [34].

Points	Percentage of Cells with Positive Reaction	Points	Color Intensity of Positive Reaction in Cells
0	0%	0	lack
1	1–10%	1	weak
2	11–50%	2	moderate
3	51–80%	3	strong
4	>80%		

**Table 2:** Clinicopathological characteristics of LSCC patients related to irisin expression.

Clinicopathological Parameter		n 140 (%)	Irisin Expression in LSCC Cancer Cells		
			Low 1–6	High >6	Chi^2^ Test *p* Value
Age *	≤60 >60	76 (56,3) 59 (43,7)	66 (47,10) 51 (36,4)	10 (7,1) 8 (5,7)	0.9457
Sex	Male Female	120 (85,7) 20 (14,3)	49 (35,0) 9 (6,4)	71 (50,7) 11 (7,8)	0.7262
Tumor size (T)	T1–T2 T3–4	54 (38,6) 86 (61,4)	50 (35,7) 70 (50,0)	4 (2,8) 16 (11,4)	0.0834
Lymph nodes * (N)	N0 N1 N2–N3	98 (72,6) 15 (11,1) 24 (17,7)	86 (61,4) 13 (9,3) 20 (14,3)	12 (8,6) 2 (1,4) 4 (2,8)	0.8475
Stage *	I II III–IV	7 (5,1) 37 (27,2) 94 (69,1)	6 (4,3) 37 (26,4) 77 (55,0)	1 (0,7) 0 (0,0) 17 (12,1)	0.0216
Grade of malignance (G)	G1 G2 G3	30 (21,5) 92 (65,7) 18 (12,8)	27 (19,3) 79 (56,4) 13 (9,3)	3 (2,1) 11 (7,8) 4 (2,8)	0.3803

Abbreviations: LSCC—laryngeal squamous cell carcinoma; significance in bold; * missing data: Age—5 cases; Lymph nodes—3 cases; Stage—2 cases.

**Table 3:** Associations of irisin expression level with clinicopathological characteristics in patients with LSCC.

LSCC	*p* Value (Mann–Whitney U Test)		Mean Value *±* SD
Lymph nodes N0 vs. N1 N0 vs. N2–3 N1 vs. N2–3	**0.0031** **0.0457** 0.5101	Lymph nodes N0 N1 N2–3	5.25 ± 2.02 3.41 ± 2.10 4.00 ± 2.68
Tumor size T1–2 vs. T3–4	**0.0348**	Tumor size T1–2 T3–4	3.00 ± 2.45 3.78 ± 2.58
Stage I vs. II I vs. III–IV II vs. III–IV	0.2785 0.7991 **0.0083**	Stage I II III–IV	3.46 ± 1.86 2.65 ± 2.52 3.83 ± 2.55
Grade of malignancy G1 vs. G2 G1 vs. G3 G2 vs. G3	0.5354 0.2857 0.4415	Grade of malignancy G1 G2 G3	3.26 ± 2.70 3.45 ± 2.47 3.95 ± 2.64

Abbreviations: LSCC—laryngeal squamous cell cancer; significance in bold.

**Table 4:** Univariate and multivariate Cox proportional hazards analyses in 140 patients with LSCC.

Clinicopathological Parameter	Univariate Analysis HR (95% CI) *p*
pT T1–T2 vs. T3–T4	0.98 (0.60–1.62) 0.9787
pN N0 vs. N+	0.76 (0.43–1.35) 0.3705
Grade G1 vs. G2–G3	0.90 (0.50–1.60) 0.7320
Stage I–II vs. III–IV	0.80 (0.48–1.33) 0.3883
Irisin <25% vs. ≥25%	1.46 (0.90–2.40) 0.1183
Ki-67 <25% vs. ≥25%	1.16 (0.68–1.98) 0.5779
MT- I/II <25% vs. ≥25%	1.18 (0.72–1.88) 0.5000
MCM3 <25% vs. ≥25%	1.37 (0.82–2.24) 0.2153
MCM5 <25% vs. ≥25 %	1.42 (0.89–2.27) 0.1442
MCM7 <25% vs. ≥25%	3.32 (0.60–18.33) 0.1665

Abbreviations: HR—hazard ratio; CI—confidence interval; LSCC—laryngeal squamous cell cancer.

## Data Availability

The raw data and the analytic methods will be made available to other researchers for purposes of reproducing the results in their own laboratories on reasonable request. To access protocols or datasets, contact katarzyna.nowinska@umw.edu.pl.
